# Highly Effective Removal of Metal Cyanide Complexes and Recovery of Palladium Using Quaternary-Ammonium-Functionalized MOFs

**DOI:** 10.3390/molecules23082086

**Published:** 2018-08-20

**Authors:** Qin Zhang, Muhan Chen, Lijiang Zhong, Qun Ye, Shaoshong Jiang, Zhangjie Huang

**Affiliations:** School of Chemistry Science and Engineering, Yunnan University, Cuihu North Road No. 2, Kunming 650091, China; 12016000408@mail.ynu.edu.cn (Q.Z.); 12015001006@ynu.edu.cn (M.C.); 12016000390@ynu.edu.cn (L.Z.); 12017000827@mail.ynu.edu.cn (Q.Y.); jiangshaosong@mail.ynu.edu.cn (S.J.)

**Keywords:** metal cyanide complexes, removal, palladium cyanide recovery, metal-organic framework

## Abstract

In this study, quaternary-ammonium-functionalized metal–organic frameworks (MOFs) Et-*N*-Cu(BDC-NH_2_)(DMF), were prepared, characterized, and applied for the highly effective removal of metal cyanide complexes, including Pd(CN)_4_^2−^, Co(CN)_6_^3^^−^, and Fe(CN)_6_^3^^−^. Batch studies were carried out, and the maximum adsorption capacities of Pd(II), Co(III), and Fe(III) reached 172.9, 101.0, and 102.6, respectively. Adsorption was rapid, and equilibrium was established within 30 min. Et-*N*-Cu(BDC-NH_2_)(DMF) exhibited high thermal and chemical stability. Furthermore, absorbed Pd(CN)_4_^2−^ was selectively recovered by two-step elution. First, Co(CN)_6_^3^^−^ and Fe(CN)_6_^3^^−^ were eluted with a 1.5 mol L^−1^ KCl solution. Elution rates of Co(CN)_6_^3^^−^ and Fe(CN)_6_^3^^−^ were greater than 98.0%, whereas the elution percentage of Pd(CN)_4_^2−^ was less than 2.0%. Second, >97.0% Pd(CN)_4_^2−^ on the loaded MOFs was eluted using a 2.0 mol L^−1^ KI solution. The recovery rate of Pd(CN)_4_^2−^ was greater than 91.0% after five testing cycles. Adsorption isotherms, kinetics models, and adsorption thermodynamics of Pd(CN)_4_^2−^ on Et-*N*-Cu(BDC-NH_2_) (DMF) were also systematically investigated. The Et-*N*-Cu(BDC-NH_2_) (DMF) absorbent exhibited a rapid, excellent ability for the adsorption of metal cyanide complexes.

## 1. Introduction

Cyanide is a highly toxic substance, and long-term exposure to low doses of cyanide can cause a significant increase in risk for skin cancer in humans, dyspnea, tachycardia, and unconsciousness. The United States, Germany, and Switzerland have proposed that the concentration of cyanide discharged into the environment should be limited to less than or equal to 0.2 mg L^−1^ [1]. Heap cyanidation has been extensively used in hydrometallurgy industries. For example, cyanide-contaminated water containing Pd(CN)_4_^2−^, Co(CN)_6_^3^^−^, and Fe(CN)_6_^3^^−^ in tailing storage facilities in the western Yunnan Province in China requires treatment [2]. Cyanide effluents from metallurgy industries have been a serious threat to the environment and public health. To protect ecological systems and human health, cyanide-containing wastewater must be treated before it is discharged into the environment. Typically, cyanides are classified as free cyanide (HCN, CN^−^) and metal cyanide complexes. Almost all the metal cyanide complexes are highly toxic to a majority of life forms. Noteworthily, biological degradation has been proven to be an environmentally benign and economically viable process for the treatment of free cyanide [3]. However, metal cyanide complexes exhibit a wide range of chemical and biological stabilities compared to free cyanide ions; therefore, they cannot be treated by biological degradation. On 30 January 2000, a dam containing toxic waste material, near Danube River in Romania, collapsed and released waste water heavily contaminated with cyanide. Moreover, metal cyanide complexes still have been reportedly observed at 2000 km away in the mouth of the Danube River [4]. 

In general, metal cyanide complexes are removed from aqueous solutions by chemical oxidation methods [5], thermal decomposition [6,7], and adsorption [8]. Slow reaction rates are observed for alkaline chlorination–oxidation, as well as cause secondary pollution to the environment. Thermal hydrolysis incurs high cost because of high pressure and high temperature requirements. Among these processes, adsorption is typically found to be the most effective approach for removing metal cyanide complexes because of its cost-effectiveness, convenience, and ecological security. Conventional adsorbents used for the removal of metal cyanide complexes include activated carbon and polymer resin [9,10]. On account of their low adsorption capacity (activated carbon), slow sorption kinetics, and relatively low thermal and chemical stability (polymer resins), their further applications on the industrial scale are limited. Thus far, the removal of metal cyanides using activated carbon or polymer resin is still at the laboratory stage [11,12]. Therefore, design and development of new sorbent materials is imperative for the highly effective adsorption and removal of metal cyanide complexes from wastewater.

In the last few years, metal–organic frameworks (MOFs) have attracted significant attention for the removal of contaminants because of their high porosity, large surface area, thermal or chemical stability, hydrophilic–lipophilic matrix, low value, and facile modification [13,14]. Specific functional groups can be introduced into the MOF matrix during synthesis to alter their sorption properties because of the combination of a highly ordered porous structure and various binding groups. Modified MOFs exhibit an excellent ability to remove various hazardous contaminants present in water, such as metal ions [15,16,17,18,19,20,21], phosphates [22,23], arsenic [24], and organic pollutants [25,26]. MOF materials have been considered as the next-generation adsorbents for the effective treatment of wastewater [27].

Furthermore, quaternary ammonium groups have been known to enhance the uptake of metal cyanide complexes [28,29,30]. As a result, the advantages of quaternary ammonium functional groups and the MOF matrix were well integrated herein. Therefore, quaternary-ammonium-functionalized MOFs can be expected to exhibit excellent adsorption properties for metal cyanide complexes. To the best of our knowledge, the use of quaternary ammonium functional groups grafted onto an MOF matrix to remove metal cyanide complexes from aqueous solutions has never been reported so far.

Among several MOFs, carboxylate multidentate ligand frameworks have been previously reported to exhibit high thermal and chemical stability [31,32,33,34,35,36]. In this study, Cu(BDC-NH_2_)(DMF) (BDC = terephthalate, DMF = *N*,*N*-dimethylformamide) was selected as the matrix for modification. The amine group on copper-based MOFs provided a platform for the introduction of hydrophobic quaternary ammonium groups into the framework of the copper-based MOFs. Quaternary ammonium was grafted onto copper-based MOFs for constructing a new adsorbent for the highly effective removal of metal cyanide complexes from aqueous solutions. Physicochemical properties of adsorbents were investigated by Fourier transform infrared (FTIR) spectroscopy, thermogravimetric analysis (TGA), scanning electron microscopy (SEM), X-ray diffraction (XRD), and N_2_ adsorption–desorption experiments. The sorption performance of activated carbon and polymer resin for metal cyanide was compared. Furthermore, the selective elution of palladium cyanide from the loaded adsorbent was also examined, which was based on a two-step elution process, and palladium cyanide was selectively recovered using a mixed solution of Co(CN)_6_^3−^, Pd(CN)_4_^2−^, and Fe(CN)_6_^3−^. Based on the experimental results and economic and environmental benefits, a novel process for the highly effective removal of metal cyanide complexes from cyanide-contaminated water was proposed. Moreover, thermodynamic parameters, adsorption isotherms, and kinetic models for palladium cyanide were also investigated. In addition, the selective recovery of palladium as a valuable by-product from cyanide wastewater was also expected.

## 2. Results and Discussion

### 2.1. Characterization

#### 2.1.1. FTIR Spectra

**Figure 1:**
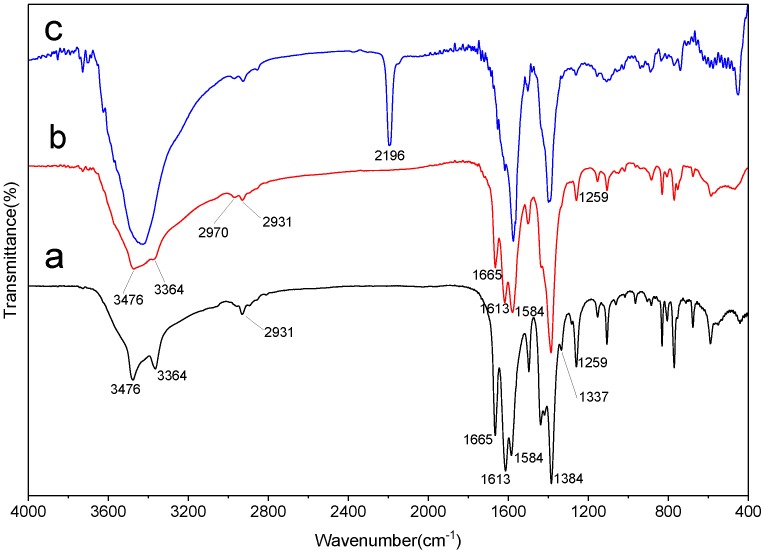
FT-IR spectra of (**a**) Cu(BDC-NH_2_)(DMF), (**b**) Et-*N*-Cu(BDC-NH_2_) (DMF) and (**c**) Et-*N*-Cu(BDC-NH_2_)(DMF)-Pd(CN)_4_^2−^.

Figure 1 shows the FTIR spectra of Cu(BDC-NH_2_)(DMF), Et-*N*-Cu(BDC-NH_2_) (DMF), and Et-*N*-Cu(BDC-NH_2_)(DMF)-Pd(CN)_4_^2−^. Double peaks are observed at 3476 and 3364 cm^−1^, corresponding to the -NH_2_ stretching vibration (Figure 1a) [37]. A sharp peak at 1665 cm^−1^ is ascribed to the C=O vibration of DMF [38]. The peak observed at 1613 cm^−1^ corresponds to the N-H bending vibration [39]. A sharp peak at 1584 cm^−1^ is attributed to the C=O bonding in the carboxylates [23], while those observed at 1337 and 1259 cm^−1^ correspond to the C-N stretching of aromatic amines [37]. Compared to those observed for pristine Cu(BDC-NH_2_)(DMF), the double peaks observed at 3476 and 3364 cm^−1^ became weak, while a small peak at 1337 cm^−1^ disappeared (Figure 1b). Furthermore, a new peak at 2970 cm^−1^ and a small peak at 2931 cm^−1^ are attributed to C-H stretching and bending of alkyl chain groups [40], indicating successful introduction of the CH_3_CH_2_- group into the Cu(BDC-NH_2_)(DMF) framework, and aromatic amines are successfully converted into quaternary ammonium group [41]. Quaternary ammonium groups provided large amount of active sites, which were favorable for the removal of metal cyanide complexes. Compared to those observed for Et-*N*-Cu(BDC-NH_2_)(DMF) (Figure 1b), characteristic peaks observed for the adsorbed species of Et-*N*-Cu(BDC-NH_2_)(DMF)-Pd(CN)_4_^2−^ do not exhibit remarkable shifts (Figure 1c), while new absorption peaks corresponding to the C≡N stretching vibration of metal cyanide complexes are observed at 2196 cm^−1^ [42]. The FTIR results also confirm the successful adsorption of metal cyanide complexes on Et-*N*-Cu(BDC-NH_2_)(DMF).

#### 2.1.2. XRD spectra

**Figure 2:**
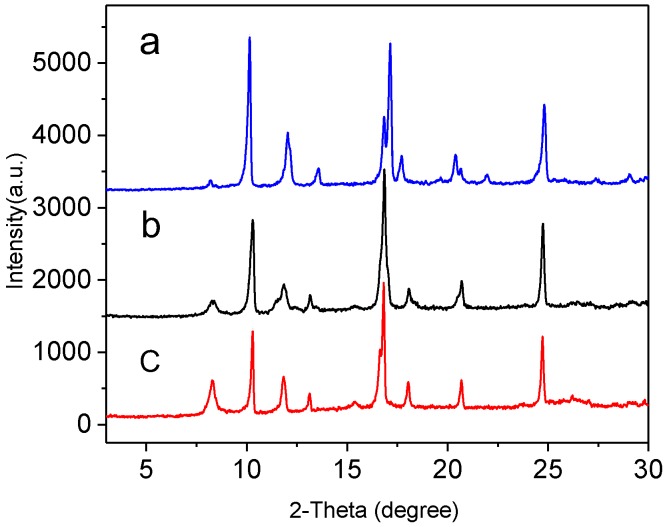
XRD patterns of (**a**) Cu(BDC)(DMF), (**b**) Cu(BDC-NH_2_)(DMF) and (**c**) Et-*N*-Cu(BDC-NH_2_)(DMF).

The XRD patterns of as-synthesized Cu(BDC)(DMF), Cu(BDC-NH_2_)(DMF), and Et-*N*-Cu(BDC-NH_2_)(DMF) are illustrated in Figure 2. The main characteristic diffraction peaks of the as-synthesized Cu(BDC)(DMF) crystals appeared at 2θ = 10.14°, 12.04°, 16.84°,17.14°, 20.38°, 20.67° and 24.80°, which is in good agreement with those simulated from the single crystal structure of Cu(BDC)(DMF) [43]. The XRD pattern of Cu(BDC)(DMF) revealed well defined double peak at 2θ = 16.84° and 17.14°. In contrast to the XRD patterns of Cu(BDC)(DMF), Cu(BDC-NH_2_)(DMF) and Et-*N*-Cu(BDC-NH_2_)(DMF) exhibited the appearance of only one peak at 2θ = 16.84° and 16.82°, respectively. Another double peak of Cu(BDC)(DMF) was observed at 2θ = 20.38° and 20.67°, while one peak at 2θ = 20.70° and 20.68° was observed for Cu(BDC-NH_2_)(DMF) and Et-*N*-Cu(BDC-NH_2_)(DMF), respectively. Furthermore, the main characteristic diffraction peaks of Cu(BDC-NH_2_)(DMF) crystals appeared at 2θ = 10.30°, 11.86°, 16.84°, 20.70°, and 24.76° (Figure 2b), which exhibited slight differences from those of Cu(BDC)(DMF). Compared to that of Cu(BDC-NH_2_)(DMF), 2θ of Et-*N*-Cu(BDC-NH_2_)(DMF) moved slightly toward small diffraction angles (Figure 2c), thus confirming that ligand functionalization does not change the original crystal structure of Cu(BDC-NH_2_)(DMF) [44,45]. Figure 2 demonstrates that both Cu(BDC-NH_2_)(DMF) and Et-*N*-Cu(BDC-NH_2_)(DMF) have very similar XRD patterns to that of the reported Cu(BDC)(DMF), confirming that the pillared three-dimensional structure is retained upon ligand functionalization [46]. Slight difference in the diffractogram was detected probably due to the variation of ligand, the flexible behavior in Cu(BDC)(DMF) structure, and the non-isotropy during sample preparation [44]. Similar quaternization reaction of the MOFs has also been reported by Wang and co-worker s [47].

#### 2.1.3. SEM Analysis

**Figure 3:**
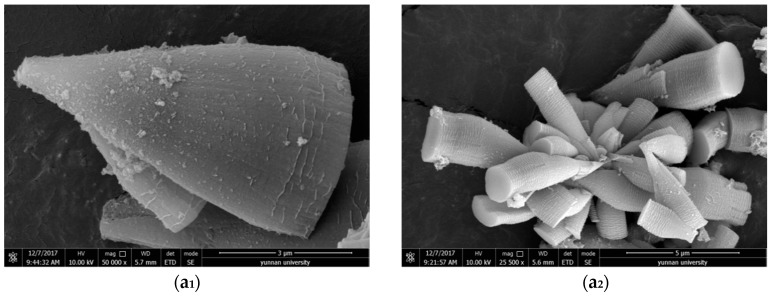

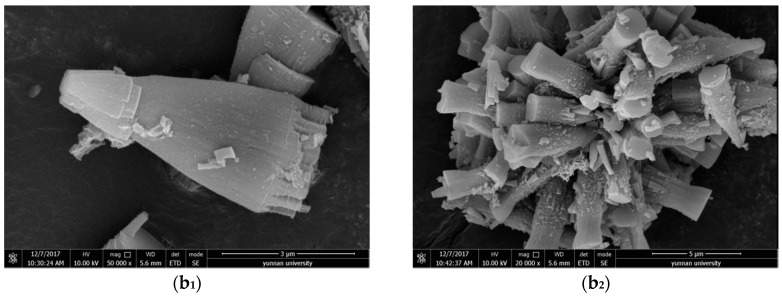
The SEM images of Cu(BDC-NH_2_)(DMF) (**a_1_**,**a_2_**) and Et-*N*-Cu(BDC-NH_2_) (DMF) (**b_1_**,**b_2_**).

To observe the surface morphology of synthesized Cu(BDC-NH_2_)(DMF) and Et-*N*-Cu(BDC-NH_2_)(DMF), SEM images were recorded. Figure 3a,b show the SEM images of Cu(BDC-NH_2_)(DMF) and Et-*N*-Cu(BDC-NH_2_)(DMF), respectively. The Cu(BDC-NH_2_)(DMF) particles exhibit gyro geometry with a regular morphology and an average particle size of ~3 μm. Compared to that observed for pristine Cu(BDC-NH_2_)(DMF), after the functionalization with quaternary ammonium group, the morphology of Et-*N*-Cu(BDC-NH_2_)(DMF) remained virtually unchanged indicating that Cu-based MOFs were successfully grafted with quaternary ammonium group without collapsing the structure [48].

#### 2.1.4. TGA

**Figure 4:**
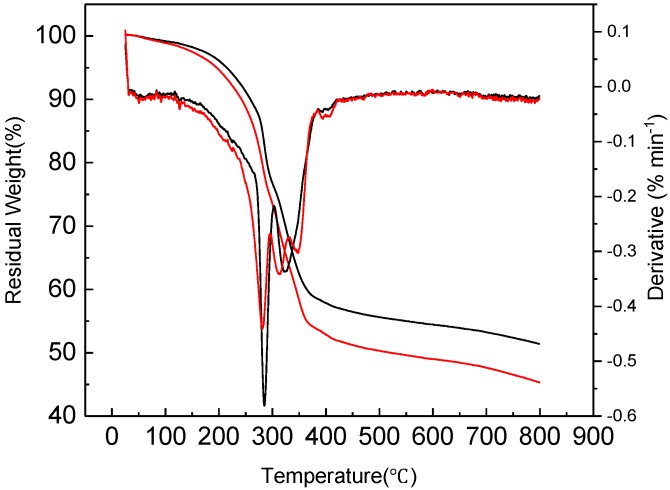
TG-DTG curves of Et-*N*-Cu(BDC-NH_2_)(DMF)(red) and Cu(BDC-NH_2_)(DMF)(black).

The thermal gravimetric analysis (TGA) spectra of Cu(BDC-NH_2_)(DMF) and Et-*N*-Cu(BDC-NH_2_)(DMF) are presented in Figure 4. The materials were heated from 8 to 800 °C at a heating rate of 10 °C min^−1^. Based on TG curves, very little difference can be observed between the behavior of Cu(BDC-NH_2_)(DMF) and Et-*N*-Cu(BDC-NH_2_)(DMF). However, the derivative thermogravimetric (DTG) results of the two samples exhibit clearly distinct profiles. The DTG curve of Cu(BDC-NH_2_)(DMF) shows two well-separated heat signals at 285 (sharp) and 325 °C (broad), corresponding to the decomposition of DMF and the framework of Cu(BDC-NH_2_)(DMF), respectively [38,45]. In contrast, decomposition of the Et-*N*-Cu(BDC-NH_2_)(DMF) is signified by three well-separated heat signals at 281, 312, and 348 °C. The two broad peaks at 312 and 348°C extending temperature range of the crystal structure collapse of Et-*N*-Cu(BDC-NH_2_)(DMF), indicating that the interaction between the quaternary ammonium molecules and the carrier could enhance the thermal stability of the Cu-MOFs to some extent [48,49]. TGA confirmed that Et-*N*-Cu(BDC-NH_2_)(DMF) possessed good thermal stability.

#### 2.1.5. N_2_ Adsorption–Desorption Isotherms

**Figure 5:**
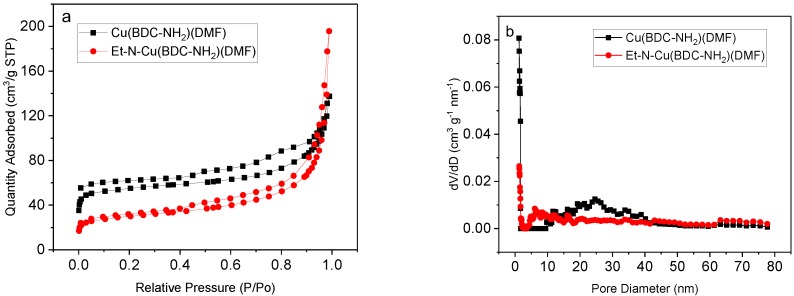
(**a**) Nitrogen adsorption–desorption isotherm of the prepared Cu(BDC-NH_2_)(DMF) and Et-*N*-Cu(BDC-NH_2_) (DMF) samples. (**b**) Pore size distributions derived from NLDFT calculations.

Figure 5a shows the N_2_ adsorption–desorption isotherms of Et-*N*-Cu(BDC-NH_2_) (DMF) and Cu(BDC-NH_2_)(DMF). The isotherms of both Cu(BDC-NH_2_)(DMF) and Et-*N*-Cu(BDC-NH_2_)(DMF) are similar to type IV isotherm. The corresponding pore-size distributions are shown in Figure 5b. As seen in Figure 5b, both meso- and micropores were clearly observed in the Cu(BDC-NH_2_)(DMF) and Et-*N*-Cu(BDC-NH_2_)(DMF) samples, respectively. Similar micro-/mesopore coexistence in Cu(BDC-NH_2_) MOFs had been reported by Morsali and co-workers [45]. Morsali et al. found that replacing BDC with BDC-NH_2_, the rate of self-assembly of Cu-MOFs was significantly interfered by amino groups, which led to the coexistence of micro-/mesopores in the as-synthesized samples. Table 1 summarizes the comparison of surface areas and total pore volume of Et-*N*-Cu(BDC-NH_2_)(DMF) and Cu(BDC-NH_2_)(DMF) with other Cu-MOFs reported in literature studies. Compared to Cu(BDC-NH_2_)(DMF), Et-*N*-Cu(BDC-NH_2_)(DMF) exhibited a decrease in both the surface area and total pore volume. The surface area dropped from 210.6 to 108.24 m^2^ g^−1^, while the total pore volume decreased from 0.37 to 0.30 cm^3^ g^-1^. The main reason for this phenomenon was that the pores of the carrier were partially occupied by the quaternary ammonium groups after modification, inferring that the quaternary ammonium functional groups existed inside the channels of the framework rather than outside the surfaces [48]. This result indicated that the quaternary ammonium group was successfully immobilized onto Cu(BDC-NH_2_)(DMF) through the quaternization reaction. Similar results have also been reported by Wang and co-workers [47]. Wang et al. found that quaternization reaction of the MOFs could significantly decrease their surface areas. Table 1 presents that the surface areas of the Cu-MOFs using terephthalate (BDC) or BDC-NH_2_ as ligand is lower than that of the benzene tricarboxylic acid (BTC) [48,50,51,52,53]. The values of the surface area of both Cu(BDC-NH_2_)(DMF) and Et-*N*-Cu(BDC-NH_2_)(DMF) are in an expected range [52,53]. The difference in the surface areas and pore size distribution of the Cu-MOFs with different ligands may be attributed to the variation in ligand, difference of the activated temperature, and the non-isotropy during sample preparation [16,44,45].

Remarkably, the specific surface areas of the samples were greater than those of a majority of the previously reported polymer resins [12,27]. The Et-*N*-Cu(BDC-NH_2_)(DMF) provided satisfactory support for the adsorption of metal cyanide complexes.

### 2.2. Effects of pH

The equilibrium loadings of Pd(CN)_4_^2−^, Co(CN)_6_^3−^, and Fe(CN)_6_^3^^−^ were examined at various pH values ranging from 7.0 to 9.0. Experiments were carried out as follows: a volume of Pd(CN)_4_^2−^, Co(CN)_6_^3−^, and Fe(CN)_6_^3^^−^ at a concentration of 100 mgL^−1^ (20 mL) was placed in three conical flasks. Then, a constant amount of Et-*N*-Cu(BDC-NH_2_)(DMF), i.e., 10 mg, was used at a temperature of 25 °C, with an adsorption time of 30 min. With the increase in the pH from 7.0 to 8.0, the equilibrium adsorption capacities (q_e_) of Pd(CN)_4_^2−^, Co(CN)_6_^3−^, and Fe(CN)_6_^3^^−^ remained almost constant, and with further increase in the solution pH, the q_e_ values of Pd(II), Co(III), and Fe(III) significantly decreased. The capacity for the adsorption of metal cyanide complexes at different pH values possibly revealed that metal cyanide complexes are adsorbed on MOFs via anion exchange [20]. In this study, adsorption was controlled at a pH of 7.0.

### 2.3. Maximum Adsorption Capacities

Tests were carried out using solutions containing only Pd(CN)_4_^2−^, Co(CN)_6_^3−^, or Fe(CN)_6_^3−^ at 25 °C. According to a previously reported method [54] the maximum adsorption capacities of Et-*N*-Cu(BDC-NH_2_)(DMF)(6d), Cu(BDC-NH_2_)(DMF), and granular activated carbon (GAC) for the metal cyanide complexes were measured. Based on the experimental data shown in Table 2, Et-*N*-Cu(BDC-NH_2_)(DMF) exhibited excellent adsorption performance toward the metal cyanide complexes compared to GAC and Cu(BDC-NH_2_)(DMF), possibly corresponding to the strong interaction between the quaternary ammonium group and the metal cyanide complexes. Quaternary ammonium grafted on Cu(BDC-NH_2_)(DMF) provided a larger amount of active sites, and adsorption was possibly explained as follows:

(1) $$\mathrm{nM}-R_{3}N^{+}I^{-}\left(S\right)+\mathrm{Me}{\left(\mathrm{CN}\right)}_{m}{}^{n-}\left(\mathrm{aq}\right)={\left(M-R_{3}N\right)}_{n}{}^{+}\mathrm{Me}{\left(\mathrm{CN}\right)}_{m}{}^{n-}\left(s\right)+{\mathrm{nl}}^{-}\left(\mathrm{aq}\right)$$

where M, S, and aq denote the MOF matrix, the Et-*N*-Cu(BDC-NH_2_)(DMF) solid surface, and the aqueous solution, respectively. From Equation (1), the main mechanism for the adsorption of metal cyanide complexes from aqueous solutions followed an ion-exchange mechanism. In addition, Van der Waals forces and electrostatic interactions played an important role in the sorption of metal ions on adsorbents [40]. In contrast, the adsorption of metal cyanide complexes by GAC and Cu(BDC-NH_2_)(DMF) mainly depended on Van der Waals forces and electrostatic interactions, respectively [27,55]. Therefore, compared to GAC and pristine Cu(BDC-NH_2_)(DMF), higher adsorption capacities for the metal cyanide complexes were achieved using Et-*N*-Cu(BDC-NH_2_) (DMF) as the adsorbent. 

### 2.4. Adsorption Kinetics

Adsorption kinetics of Pd(CN)_4_^2−^ on Et-*N*-Cu(BDC-NH_2_)(DMF)(6d) were further investigated. Experiments were carried out using single component solutions at 25 °C. Adsorption was rapid, and equilibrium was established within 30 min. Pseudo-first order, pseudo-second order, and intra-particle diffusion models were linearized as shown in Equations (2), (3), and (4), respectively, and fitted to experimental data for the adsorption of Pd(CN)_4_^2−^ on Et-*N*-Cu(BDC-NH_2_)(DMF). Table 3 summarizes the model data for Pd(CN)_4_^2−^:

(2) $$\mathrm{lg}\left(q_{e}-q_{t}\right)={\mathrm{lgq}}_{e}-\frac{k_{1}t}{2.303}$$

(3) $$\frac{t}{q_{t}}=\frac{1}{k_{2}{q_{e}}^{2}}+\frac{t}{q_{e}}$$

(4) $$q_{t}=k_{p}t^{1/2}+C$$

where, q_e_ and q_t_ represent the loading of Pd(II) at equilibrium and at time t, respectively; k_1_ is the pseudo-first-order constant; k_2_ is the pseudo-second-order constant; kp is the intraparticle diffusion rate constant; and C (mg g^−1^) is the boundary layer thickness.

**Figure 6:**
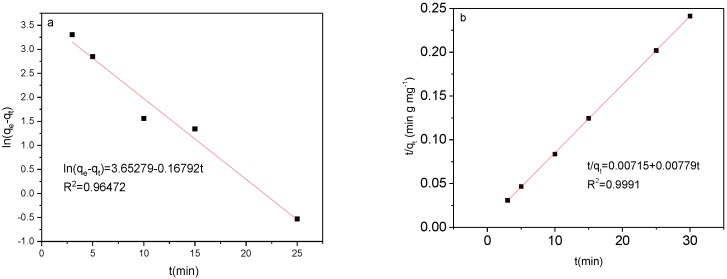

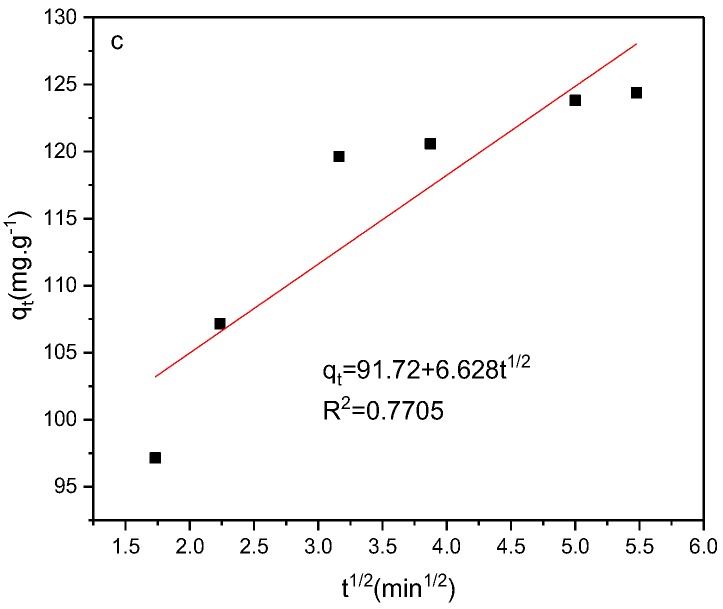
(**a**) Pseudo-first-order, (**b**) pseudo-second-order and (**c**) intra-particle diffusion model at 298 K.

The adsorption kinetics for Pd(II) fitted well the pseudo-second-order kinetic model. On the other hand, poor correlation coefficients were obtained for the pseudo-first-order and intraparticle diffusion models (Figure 6). Adsorption kinetics revealed that chemical sorption is likely the rate-limiting step for the adsorption of Pd(II) on the Et-*N*-Cu(BDC-NH_2_)(DMF) adsorbent. Previously, the adsorption of Pd(CN)_4_^2^^−^ on the polymer resin or activated carbon was quite slow, taking more than 8 and 2 h to reach adsorption equilibria, respectively [9,12,56]. Compared with the adsorption equilibria using the polymer resin and activated carbon, the adsorption equilibrium of Pd(II) using Et-*N*-Cu(BDC-NH_2_)(DMF) was more rapid. On account of the unique mesoporous structure and larger quantity of polar groups of Et-*N*-Cu(BDC-NH_2_)(DMF), a high affinity was observed between metal cyanide complexes and the MOFs. Hence, Pd(CN)_4_^2−^ rapidly spreads into the MOF matrix. A rapid adsorption equilibrium for Pd(CN)_4_^2−^ was expected. In contrast, a hydrophobic structure for polystyrene–divinylbenzene matrices lead to a lower affinity between polymer resins and metal cyanide complexes. Transport rates of Pd(CN)_4_^2−^ on polystyrene–divinylbenzene matrices were less than that on Et-*N*-Cu(BDC-NH_2_)(DMF). An extremely long time was taken to attain adsorption equilibrium on activated carbon, corresponding to the slow diffusion of Pd(CN)_4_^2−^ from the internal pores during transportation to the adsorbent surfaces [57]. 

### 2.5. Sorption Isotherms

**Figure 7:**
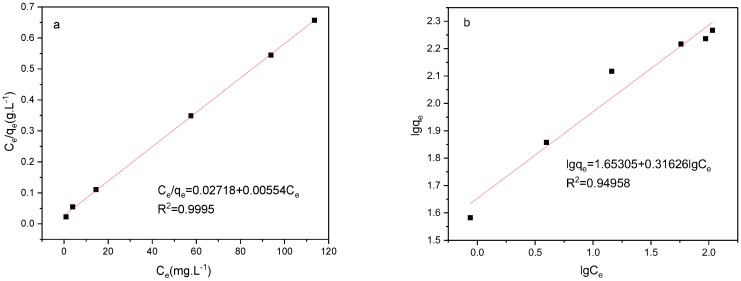
(**a**) Langmuir and (**b**) Freundlich isotherm models.

Sorption isotherms of Pd(CN)_4_^2−^ on Et-*N*-Cu(BDC-NH_2_)(DMF) (6d) were also further investigated using single component solutions at 25 °C. A fixed amount of Et-*N*-Cu(BDC-NH_2_)(DMF) (10 mg) was added to a series of 150-mL conical flasks with Pd(CN)_4_^2−^ solutions (20 mL, 20–200 mg L^−1^, pH = 7.0). Then, the conical flasks were placed on a shaker at 130 rpm, and the temperature was maintained at 25 °C for 30 min. Experimental data were fitted with the Langmuir and Freundlich equilibrium models as follows:

(5) $$\frac{c_{e}}{q_{e}}=\frac{1}{q_{m}b}+\frac{c_{e}}{q_{m}}$$

(6) $$lgq_{e}=lgk_{F}+\frac{1}{n}lgC_{e}$$

where, q_m_ is the maximum adsorption capacity; b is the Langmuir adsorption equilibrium constant; K_F_ is the Freundlich constant; and 1/n is the adsorption intensity. Table 4 and Figure 7 show the results obtained.

Experimental data revealed that the adsorption Pd(CN)_4_^2−^ on Et-*N*-Cu(BDC-NH_2_) (DMF) well conforms to the Langmuir equation. The q_m_ value for the adsorption of Pd(CN)_4_^2−^ on the Et-*N*-Cu(BDC-NH_2_) (DMF) adsorbent was calculated to be 180.5 mg g^−1^ from the Langmuir isotherm model at 25 °C. This value is in good agreement with that obtained from experimental data (Table 4).

### 2.6. Thermodynamic Parameters

The thermodynamic equilibrium constant K_c_ for adsorption was calculated according to the following equation:

(7) $$K_{c}=\frac{{(C}_{o}-C_{e})V}{{\mathrm{MC}}_{e}}$$

Here, C_o_, C_e_, V and M represent the initial concentration; the equilibrium concentration; Pd(CN)_4_^2−^ solution volume; and Et-*N*-Cu(BDC-NH_2_)(DMF) mass(6d), respectively. Enthalpy changes (△H) and entropy changes (△S) were obtained from the vant Hoff equation:

(8) $${\mathrm{lnK}}_{C}=-\frac{△H}{\mathrm{RT}}+\frac{△S}{R}$$

**Figure 8:**
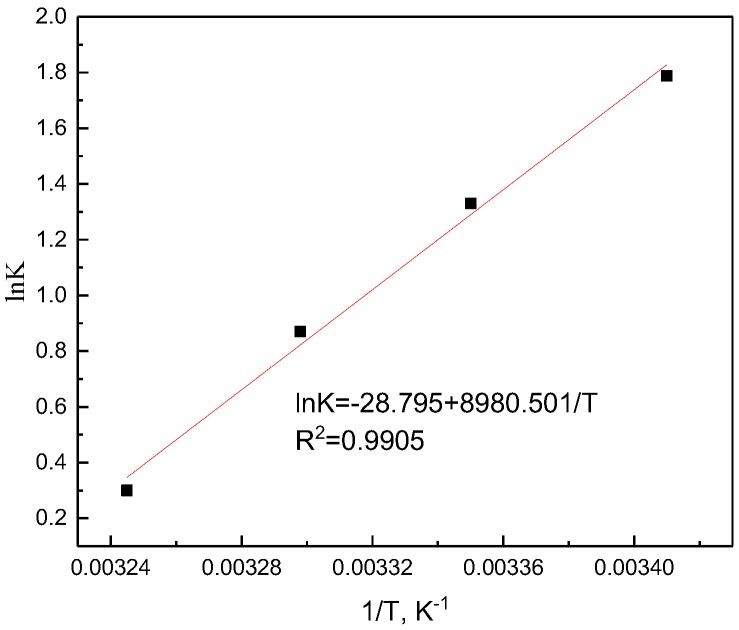
Van’t Hoff plot for the investigated adsorbents.

Figure 8 plots lnKc versus 1/T. △H and △S values were calculated from the slope and intercept of the vant Hoff plot, respectively; furthermore, the Gibbs free-energy changes (△G) were calculated using the following equation:

(9) △G=△H−T△S

Table 5 shows the results. △G values were −4.48, −3.29, −2.09, and −0.89 kJ mol^−1^ at 293.15, 298.15, 303.15, and 308.15 K, respectively, indicating that the degree of a spontaneous reaction decreases with increasing temperature. Hence, adsorption is considerably favorable at a low temperature. A negative △H (−74.66 kJ mol^−1^) value confirmed that the reaction is exothermic, while a negative △S values implies the decrease in randomness at the interface between Et-*N*-Cu(BDC-NH_2_)(DMF) and the solution during the adsorption of Pd(CN)_4_^2−^ on Et-*N*-Cu(BDC-NH_2_)(DMF). A mixed physicochemical process is the most widely accepted mechanism for the adsorption of metal ions on various materials [40]. Under normal conditions, the △H value for chemisorption was greater than 40 kJ mol^−1^ [58]. Based on the experimental data in Table 3, chemisorption is possibly the rate-controlling step for the adsorption of Pd(CN)_4_^2−^ on Et-*N*-Cu(BDC-NH_2_)(DMF).

### 2.7. Removal of Metal Cyanide Complexes and Recovery of Pd(II)

Et-*N*-Cu(BDC-NH_2_)(DMF) (6d) was applied for the removal of metal cyanide complexes from a mixed solution. Typically, 50 mg of the adsorbent was added to the mixed solutions of Pd(CN)_4_^2−^, Co(CN)_6_^3−^, and Fe(CN)_6_^3^^−^. The total volume of the mixed solutions was 100 mL, and the concentrations of Pd(II), Co(II), and Fe(III) in mixed solutions were 50.1, 25.3, and 25.4 mg L^−1^, respectively. Batch adsorption experiments were carried out by the same sorption procedure as that described above. Table 6 shows the experiment results. From Table 6, 99.1% of Pd(II), 98.7% of Co(II), and 98.3% Fe(III) were adsorbed on Et-*N*-Cu(BDC-NH_2_)(DMF). Then, two-step elution was designed to elute the metal cyanide complexes loaded on Et-*N*-Cu(BDC-NH_2_)(DMF). First, Co(CN)_6_^3^^−^ and Fe(CN)_6_^3^^−^ were eluted using a 1.5 mol L^−1^ KCl solution, followed by the elution of Pd(CN)_4_^2−^from Et-*N*-Cu(BDC-NH_2_)(DMF) using 2.0 mol L^−1^ KI solutions. Batch elution experiments were carried out. Based on the experimental data in Table 6, in the first step, when KCl solutions were used as the eluent, the elution ratios of Co(CN)_6_^3^^−^ and Fe(CN)_6_^3^^−^ was greater than 98.2%, whereas that of Pd(CN)_4_^2−^ was less than 2.0%. In the second step, the elution percentage of Pd(II) reached 97.2% using the KI solution. The recovery rate of Pd(CN)_4_^2−^ was greater than 96.0%. The study results demonstrated that Et-*N*-Cu(BDC-NH_2_)(DMF) can be used for the highly effective removal of metal cyanide complexes from aqueous solutions. Furthermore, Pd(CN)_4_^2−^ loaded on Et-*N*-Cu(BDC-NH_2_)(DMF) can be selectively separated during the elution in two steps using different eluting agents.

Based on the principle of the minimum charge density, the charge density of Pd(CN)_4_^2−^ is less than those of Co(CN)_6_^3−^ and Fe(CN)_6_^3−^. Fewer water molecules are required to stabilize Pd(CN)_4_^2−^ compared to multivalent anions in the aqueous solution [59,60]. Pd(CN)_4_^2−^ possibly exhibited higher affinity for hydrophobic quaternary ammonium compared to Co(CN)_6_^3−^ or Fe(CN)_6_^3−^. Therefore, Co(CN)_6_^3−^ and Fe(CN)_6_^3−^ adsorbed on Et-*N*-Cu(BDC-NH_2_)(DMF) can be eluted more easily compared to Pd(CN)_4_^2−^. The size of I^−^ is well known to be greater than that of Cl^−^, leading to the lower charge density of I^−^ compared to Cl^−^. Therefore, the interaction of I^−^ with $M-R_{3}N^{+}$ is considerably greater than that of Cl^−^, which was completely in conformity with the “perchlorate effect” [29]. Experimental results revealed that Pd(CN)_4_^2−^ adsorbed on Et-*N*-Cu(BDC-NH_2_)(DMF) can be completely eluted with KI. The elution reaction for the halide ion might occur as follows:

(10) $$\left(M-R_{3}N\right){)}_{n}{}^{+}\mathrm{Me}{\left(\mathrm{CN}\right)}_{m}{}^{n-}\left(s\right)+{\mathrm{nX}}^{-}\left(\mathrm{aq}\right)=\mathrm{nM}-R_{3}N^{+}X^{-}\left(S\right)+\mathrm{Me}{\left(\mathrm{CN}\right)}_{m}{}^{n-}\left(\mathrm{aq}\right)$$

where, M, S, X, and aq denote the MOF matrix, the Et-*N*-Cu(BDC-NH_2_) (DMF) solid surface, halide (Cl or I), and the aqueous solution, respectively. From Equation (10), the stronger the interaction between $\left(M-R_{3}N\right){)}_{n}{}^{+}$ and the halide anion, the higher the elution rate. The elution of metal cyanide complexes from Et-*N*-Cu(BDC-NH_2_)(DMF) followed an ion-exchange mechanism.

### 2.8. Chemical Stability and Regeneration Experiment

**Figure 9:**
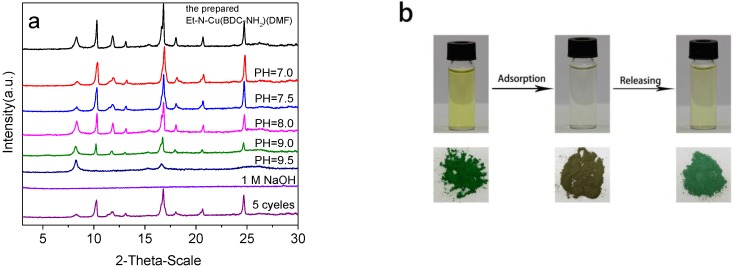
(**a**) The XRD patterns of Et-*N*-Cu(BDC-NH_2_)(DMF) (6d) at different pH values, compared with the prepared one (Room temperature). pH = 7.0 (24 h), pH = 7.5 (24 h), pH = 8.0 (24 h), pH = 9.0 (2 h), pH = 9.5 (1 h), 5 cycles (pH = 7.0), 1 mol L^−1^ NaOH (1 h). (**b**) The photographs show adsorption and releasing metal cyanide complexes using Et-*N*-Cu(BDC-NH_2_)(DMF) adsorbent.

To investigate the chemical stability of the material, Et-*N*-Cu(BDC-NH_2_)(DMF) was first suspended in aqueous solutions at different pH, which was followed by characterization by XRD patterns to monitor the changes of the crystallinity of the MOFs. Figure 9a demonstrates that the crystallinity of Et-*N*-Cu(BDC-NH_2_)(DMF) (6d) does not show significant lose at various pH values ranging from 7.0 to 8.0 (room temperature). After five adsorption-desorption cycles at pH = 7.0, the crystallinity of the sample was only partially decomposed. With further increase in the pH of the solution, the MOF was partially decomposed. The original crystallinities of the structures were completely destroyed, and the sample underwent complete amorphization in NaOH solutions (1.0 mol L^−1^). It was found that the prepared Et-*N*-Cu(BDC-NH_2_)(DMF) was unstable under strongly basic conditions and dissolved gradually.

Various independent factors play a critical role in the water stability of MOFs, for example, metal-type, metal ligand coordination environment, steric factors, topology, and porosity [61]. Some functionalized Cu(BDC) MOFs materials have been used as adsorbent or catalyst in aqueous solutions. For example, Rahmani et al. reported that Cu(BDC)(DMF) could be used as a stable catalyst for the reduction of methyl orange with sodium borohydride (NaBH_4_) in aqueous solutions [62]. Gong and group found that Cu(BDC-NH_2_) (4,4’-Bipy) could be used as adsorbent to adsorb methyl violet in basic water (pH = 9) [53]. In this study, quaternary ammonium salt-functionalized Cu(BDC-NH_2_)(DMF) MOFs showed high water stability in the neutral and weakly basic aqueous solutions.

**Figure 10:**
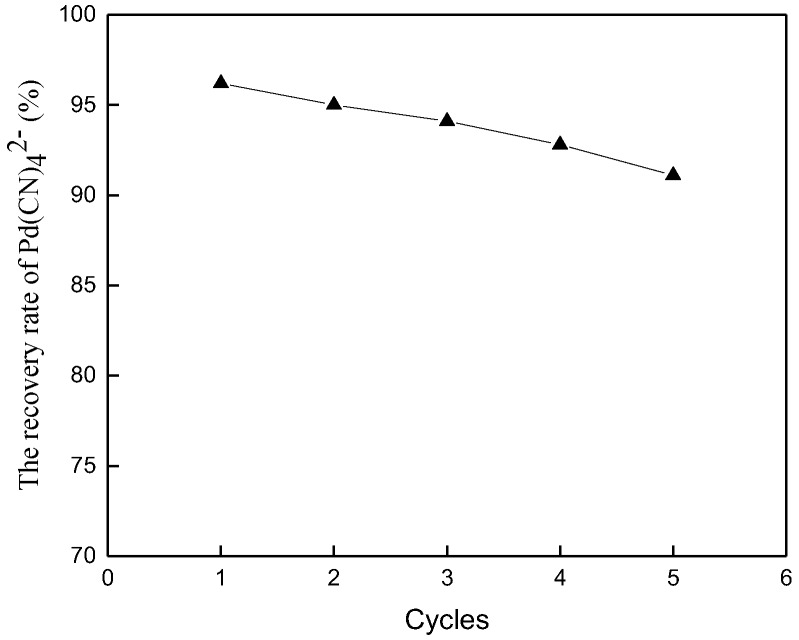
The recovery rates for Pd(CN)_4_^2−^ in the mixed solution for five cycles.

To evaluate the regeneration ability of Et-*N*-Cu(BDC-NH_2_)(DMF), the maximum adsorption capacities of five adsorption–desorption cycles in single Pd(CN)_4_^2−^ solutions were estimated. The loss of the maximum adsorption capacities was less than 5% after five cycles. The separation of Pd(CN)_4_^2−^ from a mixed solution containing Co(CN)_6_^3−^ and Fe(CN)_6_^3−^ was also carried out. The corresponding recovery rates of Pd(CN)_4_^2−^ for all five cycles were greater than 91.0% (Figure 10). According to the experimental results, the Et-*N*-Cu(BDC-NH_2_)(DMF) adsorbent exhibited efficient removal and separation of metal cyanide complexes from the neutral and weak base aqueous solutions, as well as good stability and reusability. 

In comparison with activated carbon and polymer resin, Et-*N*-Cu(BDC-NH_2_)(DMF) exhibit many advantages, for example, quick sorption kinetics, high adsorption capacity, and high selectivity (Table 7).

## 3. Conclusions

In this study, quaternary-ammonium-functionalized MOFs were synthesized for removal of metal cyanide complexes from the neutral and weakly basic aqueous solutions in batch-type experiments. Et-*N*-Cu(BDC-NH_2_)(DMF) was easily synthesized from Cu(BDC-NH_2_)(DMF) using commercially available reagents. The prepared Et-*N*-Cu(BDC-NH_2_)(DMF) absorbent was well characterized by FTIR, TGA, SEM, XRD, and N_2_ adsorption–desorption studies. The unique matrix structure and abundant active adsorption sites led to high removal efficiencies for Pd(CN)_4_^2−^, Co(CN)_6_^3−^, and Fe(CN)_6_^3−^ from aqueous solutions. The sorption kinetics for the sorption of Pd(CN)_4_^2−^ on Et-*N*-Cu(BDC-NH_2_)(DMF) were well fitted by a pseudo-second-order model, while the Langmuir model well described the sorption isotherms. The thermodynamics parameter values for △H, △S, and △G were also estimated. Furthermore, adsorbed Pd(CN)_4_^2−^ was selective recycled by two-step elution. The Et-*N*-Cu(BDC-NH_2_)(DMF) absorbent demonstrated immense potential for the treatment of metal cyanide complexes from cyanide-contaminated water.

## 4. Experimental

### 4.1. Reagents and Instruments

**Scheme 1:**
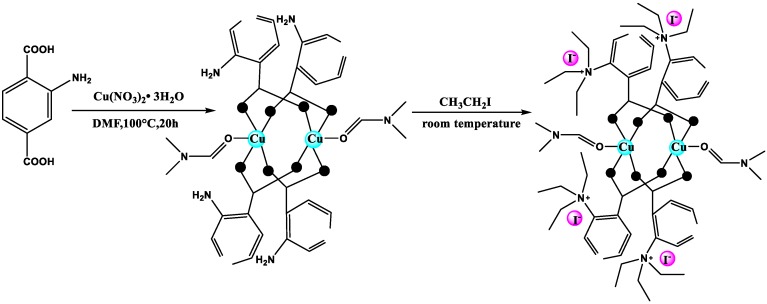
Synthesis of Et-*N*-Cu(BDC-NH_2_) (DMF) adsorbents.

Scheme 1 shows the two-step preparation of the target adsorbents. First, Cu(BDC-NH_2_)(DMF) was obtained by a solvothermal method [41]. In a typical synthesis, Cu(NO_3_)_2_·3H_2_O (0.968 g, 4 mmol) and 2-aminoterephthalic acid (0.0905 g, 0.5 mmol) were added to a *N*,*N*-dimethylformamide (DMF) solution (50 mL) and placed in an autoclave. The resulting mixture was heated at 100 °C for 20 h. The final product was filtered and washed twice with CHCl_3_ and dried under vacuum at 120°C for 10 h. Second, Cu(BDC-NH_2_)(DMF) (0.03 g) was added into dry DMF (3 mL) containing three equivalents of ethyl iodide (EtI) at room temperature for 6 days. The solid thus obtained was washed with CHCl_3_ and dried overnight in a vacuum oven at 130 °C for 24 h. The resulting powder was denoted as Et-*N*-Cu(BDC-NH_2_)(DMF). 

**Figure 11:**
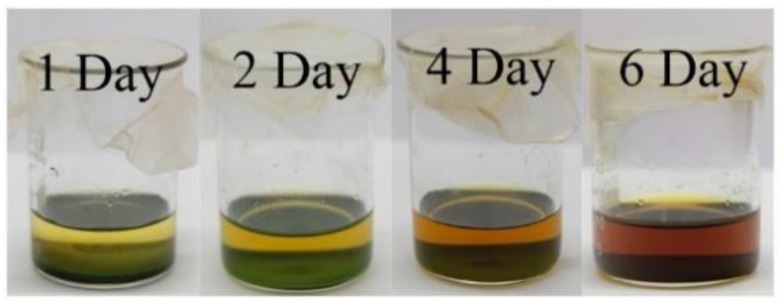
Quaternization reaction from Cu(BDC-NH_2_)(DMF) to Et-*N*-Cu (BDC-NH_2_) (DMF).

To date, thermal activation is the most straightforward way to remove coordinated solvent molecules. Besides, for the exchange of pore-filling solvents in MOFs, chemical activation is also widely used in recent several years [64,65]. In this study, *N*,*N*-dimethylformamide (DMF) was selected as a solvent for the MOF synthesis because of its high boiling point and ability to dissolve carboxylic acids and metal salts. Bordiga et al. reported the effect of temperature on the removal of DMF from the structure. X-ray diffraction (XRD) analysis clearly indicated that for the sample activated at 225 °C the characteristic peaks of Cu(BDC)(DMF) still existed [43]. Therefore, after higher temperature activation (130 °C) DMF was not removed. Et-*N*-Cu(BDC-NH_2_)(DMF) was synthesized according to Scheme 1 [41]. The color of solution burn with prolonging the reaction time of functionalization, indicating that the conversion yield from Cu(BDC-NH_2_)(DMF) to Et-*N*-Cu(BDC-NH_2_)(DMF) increased (Figure 11). In order to test the yield of quaternization reaction, Et-*N*-Cu(BDC-NH_2_)(DMF) was digested using dilute hydrochloric acid and the iodine content of the sample was analyzed [47]. The result of the iodine content in the framework of Et-*N*-Cu(BDC-NH_2_)(DMF), indicated that about 14, 26, and 32% of -NH_2_ group was converted to the group of quaternary ammonium salt for Et-*N*-Cu(BDC-NH_2_)(DMF)-2d, Et-*N*-Cu(BDC-NH_2_)(DMF)-4d, Et-*N*-Cu(BDC-NH_2_)(DMF)-6d, respectively(Table 8). Et-*N*-Cu(BDC-NH_2_)(DMF)-6d was used as adsorbent for removal of metal cyanide complexes. The results are summarized in Table 8.

Activated carbon and Cu(NO_3_)_2_·3H_2_O were purchased from Alfa Aesar (Beijing, China). Pd(CN)_4_^2−^, Co(CN)_6_^3−^, Fe(CN)_6_^3^^−^, and 2-aminoterephthalic acid were purchased from Sigma-Aldrich (Saint Louis, MO, USA). The concentrations of all metal ions were determined on an ICP-AES instrument (ICAP 6300, Thermo Fisher Scientific, Waltham, MA, USA). The phase composition of adsorbents was determined by XRD (Shimadzu, Kyoto, Japan) over a 2*θ* range from 5° to 30°. FTIR spectra of Cu-based MOFs were recorded on a Fourier transform infrared spectrometer (Thermo Nicolet Corp., Waltham, MA, USA). Surface areas of Cu-based MOFs were measured by nitrogen adsorption–desorption isotherms at 77 K on a Micromeritics Tristar apparatus (Micromeritics Instrument Corporation, Norcross, GA, USA). Thermal properties of the samples were investigated by TGA (SDT-Q600, TA Instruments, New Castle, DE, USA) at 8–800 °C under nitrogen.

### 4.2. Adsorption Experiments

Batch adsorption experiments were carried out to examine the removal of metal cyanide complexes from aqueous solutions. Typically, adsorption experiments were carried out at 25 °C. An adsorption solution 20 mL for each of Pd(CN)_4_^2−^, Co(CN)_6_^3−^, and Fe(CN)_6_^3^^−^ at concentrations of 100 mgL^−1^ was added in three sealed conical flasks. Then, 10 mg of the adsorbent(Et-*N*-Cu(BDC-NH_2_) (DMF)-6d) was added to the Pd(CN)_4_^2−^, Co(CN)_6_^3−^, and Fe(CN)_6_^3^^−^ solutions. Subsequently, the conical flasks were placed on a thermostatic shaker for 30 min at 130 rpm. Then, the mixture was subjected to centrifugation for 5 min at 5000 rpm. The metal ions in the solution were estimated by ICP-AES (ICAP 6300, Thermo Fisher Scientific, Waltham, MA, USA).

## Figures and Tables

**Table 1:** Comparison of surface areas and total pore volums of Et-*N*-Cu(BDC-NH_2_)(DMF) and Cu(BDC-NH_2_)(DMF) with other Cu-MOFs reported in literature studies.

Sample	Surface Area of BET (m^2^ g^−1^)	Total Pore Volume (cm^3^ g^−1^)	Ref.
Cu-BTC	692–2211	~0.81	[48,50,51,52]
Cu(BDC)(DMF)	241–903	~0.30	[52]
Cu(BDC-NH_2_)(4,4’-Bipy)_0.5_	124	-	[53]
Cu(BDC-NH_2_)(DMF)	210.6	0.37	present work
Et-*N*-Cu(BDC-NH_2_)(DMF)	108.24	0.30	present work

**Table 2:** The maximum adsorption capacities for the metal cyanide complexes.

Metal Cyanide Complexes	q_max_, Et-*N*-Cu(BDC-NH_2_) (DMF)	q_max_, Cu(BDC-NH_2_) (DMF)	q_max_, GAC
Pd(II)	172.9	37.8	18.2
Co(III)	101.0	13.9	18.1
Fe(III)	102.6	20.6	20.7

**Table 3:** Kinetic parameters of Pd(II) adsorption onto the Et-*N*-Cu(BDC-NH_2_)(DMF) adsorbents.

Pseudo-First-Order Model		Pseudo-Second-Order Model		Intra-Particle Diffusion Model	
q_e_, exp. (mg g^−1^)	124.4	q_e_, exp. (mg g^−1^)	124.4	Kp (mg g^−1^ min^−0.5^)	6.628
q_e_, cal. (mg g^−1^)	38.58	q_e_, cal. (mg g^−1^)	128.4	C	91.72
k_1_ (min^−1^)	0.17	k_2_ (g mg^−1^ min^−1^)	0.008	R^2^	0.7705
R^2^	0.9647	R^2^	0.9991		

**Table 4:** Langmuir and Freundlich isotherm parameters of Et-*N*-Cu(BDC-NH_2_)(DMF).

Metal	q_m_^a^ (mg g^−1^)	Langmuir Isotherm			Freundlich Isotherm		
		q_m_^b^ (mg g^−1^)	B (L mg^−1^)	R^2^	K_f_ (L g^−1^)	n	R^2^
Pd	172.9	180.5	0.213	0.9995	44.98	3.16	0.9496

^a^ Experimental data. ^b^ Calculated value according to Langmuir isotherm model.

**Table 5:** Thermodynamic parameters for the absorption of Pd(CN)_4_^2−^.

Temperature (K)	△G (kJ mol^−1^)	△S (J mol^−1^ K^−1^)	△H (kJ mol^−1^)
293.15	−4.48	−239.4	−74.7
298.15	−3.29		
303.15	−2.09		
308.15	−0.89		

**Table 6:** Adsorption and separation of metal cyanide complexes from mixed solution.

Metal Cyanide Complexes	Pd(CN)_4_^2−^	Co(CN)_6_^3−^	Fe(CN)_6_^3−^
Initial concentration (mg L^−1^)	50.1	25.3	25.4
Adsorption rate (%)	99.1	98.7	98.3
The first elution rate (%)	1.9	98.2	98.8
The second elution rate (%)	97.2		
Recovery rate (%)	96.3		

**Table 7:** Comparison of the maximum adsorption capacity, adsorption time, and adsorption selectivity for Pd(CN)_4_^2−^ with polymer resins and activated carbon.

Adsorbent	Capacity (mg g^−1^)	Adsorption Time (h)	Optimum pH	Adsorption Selectivity	Refs.
Polymer resins	<80	8–48 h	9–11	Pd(CN)_4_^2−^ coexisting with other metal cyanide	[10,11,12,27]
Activated carbon	8–25	2–12 h	9–11	Pd(CN)_4_^2−^ coexisting with other metal cyanide	[3,4,5,9,55,63]
Et-*N*-Cu(BDC-NH_2_) (DMF)	>100	<0.5	7–8	Pd(CN)_4_^2−^ can eluted separation from other metal cyanide	Present work

**Table 8:** The reaction yield from Cu(BDC-NH_2_)(DMF) to Et-*N*-Cu(BDC-NH_2_)(DMF).

Samples	Iodine Content (w%)	Reaction 7ield (%)
Et-*N*-Cu(BDC-NH_2_) (DMF)-2d	3.5	~14
Et-*N*-Cu(BDC-NH_2_) (DMF)-4d	6.3	~26
Et-*N*-Cu(BDC-NH_2_) (DMF)-6d	7.8	~32
